# Supplementary material for: Exploring the Role of Inflammation and Metabolites in Bell’s Palsy and Potential Treatment Strategies
Source: Biomedicines. 2025 Apr 13;13(4):957. doi: 10.3390/biomedicines13040957 (PMC12024589; doi:10.3390/biomedicines13040957)
Supplement: Supplementary file 1 [file biomedicines-13-00957-s001.zip › STROBE-MR checklist.pdf]

STROBE-MR checklist of recommended items to address in reports of Mendelian randomization studies[1 2]

Item No.	Section		Checklist item	Page No.	Relevant text from manuscript
1	**TITLE and ABSTRACT**		Indicate Mendelian randomization (MR) as the study's design in the title and/or the abstract if that is a main purpose of the study	1-2	Exploring the Relationship Between Proton Pump Inhibitors Target Genes, inflammatory proteins and Asthma: A Mendelian randomization study with mediation analysis
	**INTRODUCTION**				
2	**Background**		Explain the scientific background and rationale for the reported study. What is the exposure? Is a potential causal relationship between exposure and outcome plausible? Justify why MR is a helpful method to address the study question	3	This study uses Mendelian randomization to explore if there's a causal link between genes targeted by proton pump inhibitors (PPIs) and asthma. By leveraging genetic variants as instrumental variables, the research aims to overcome confounding issues present in observational studies, providing clearer insights into the potential effects of PPIs on asthma risk.
3	**Objectives**		State specific objectives clearly, including pre-specified causal hypotheses (if any). State that MR is a method that, under specific assumptions, intends to estimate causal effects	3	The study's objective is to determine whether PPI target genes causally influence asthma risk. It hypothesizes that genetic variations affecting these genes' expression will reveal a causal relationship with asthma. MR, under assumptions like IV validity and no horizontal pleiotropy, is used to estimate these potential causal effects by leveraging genetic proxies for PPI exposure.
	**METHODS**				
4	**Study design and data sources**		Present key elements of the study design early in the article. Consider including a table listing sources of data for all phases of the study. For each data source contributing to the analysis, describe the following:		
		a)	Setting: Describe the study design and the underlying population, if possible. Describe the setting, locations, and relevant dates, including periods of recruitment, exposure, follow-up, and data collection, when available.	4-5	The study employs a Mendelian randomization design leveraging genetic data from the eQTLGen consortium and GTEx project as instrumental variables. It involves a virtual population derived from these genetic databases, without specific geographic locations or recruitment periods. Data collection is based on publicly available datasets, focusing on gene expression and asthma outcomes, with no direct patient interaction or follow-up. The analysis timeframe aligns with the availability of these genetic datasets, reflecting a

				contemporary analysis rather than a longitudinal study.
	b)	Participants: Give the eligibility criteria, and the sources and methods of selection of participants. Report the sample size, and whether any power or sample size calculations were carried out prior to the main analysis	4-5	Participants in this study are inferred from genetic data sources such as the eQTLGen consortium and GTEx project, with no specific eligibility criteria or selection methods as it involves a genetic analysis rather than direct participant recruitment. The sample size is determined by the available genetic data within these databases. Power or sample size calculations are not typically conducted in MR studies using existing genetic datasets, as the analysis relies on the data available in these repositories. The study leverages summary statistics from genome-wide association studies (GWAS) to infer causal associations.
	c)	Describe measurement, quality control and selection of genetic variants	5	The study utilizes genetic variants from the eQTLGen consortium and GTEx project as instrumental variables (IVs) to proxy for the expression of PPI target genes. The selection of genetic variants involves stringent quality control measures to ensure the variants are significantly associated with gene expression (minor allele frequency > 1%, p < 1×10^-5). The variants are further refined through linkage disequilibrium clustering using PLINK, pruning genetic variants in high linkage disequilibrium (r^2 > 0.1 within a 10,000 kb window) to remove multicollinearity. F-statistics are calculated to exclude weak instruments, with a threshold of 10 to ensure the strength of the IVs. The process aims to select robust genetic variants that meet the criteria for valid Mendelian randomization analysis.
	d)	For each exposure, outcome, and other relevant variables, describe methods of assessment and diagnostic criteria for diseases	5	In this study, exposures are genetic variants associated with PPI target genes, serving as instrumental variables. Outcomes, such as asthma, are identified through consortium databases like TAGC and FinnGen, using established diagnostic criteria or ICD codes. Other variables, including inflammatory proteins, are sourced from the EBI GWAS Catalog, with assessments based on standardized immunological measures.
	e)	Provide details of ethics committee approval and participant informed consent, if relevant	5	As this study is based on secondary analysis of publicly available genetic databases, it did not require ethics committee approval or participant

					informed consent. Original data collection by consortiums like eQTLGen, GTEx, and FinnGen would have obtained necessary ethical approvals and consents.
5	**Assumptions**		Explicitly state the three core IV assumptions for the main analysis (relevance, independence and exclusion restriction) as well assumptions for any additional or sensitivity analysis	6	In this Mendelian randomization study, the core assumptions are that the genetic variants (IVs) are relevant to the expression of PPI target genes, independently associated with asthma only through these genes, and free from direct effects on asthma outside this pathway. Sensitivity analyses verify these assumptions, ensuring the robustness of the causal inferences drawn between PPI target genes and asthma risk.
6	**Statistical methods: main analysis**		Describe statistical methods and statistics used		
		a)	Describe how quantitative variables were handled in the analyses (i.e., scale, units, model)	5	The study employs quantitative genetic data from eQTLGen and GTEx, where gene expression levels are typically reported as continuous variables. These expressions are scaled and processed to fit within the analytical models used for Mendelian randomization. The models, such as inverse variance weighted (IVW) and MR-Egger, handle these variables to estimate the causal effects on asthma risk, considering the genetic variants as instrumental variables. Units are standardized within the dataset, and the analysis models adjust for potential confounders to isolate the effect of gene expression on asthma outcomes.
		b)	Describe how genetic variants were handled in the analyses and, if applicable, how their weights were selected	5	In the analyses, genetic variants were utilized as instrumental variables (IVs) to proxy the effect of proton pump inhibitor (PPI) target gene expression on asthma risk. These variants were selected based on their significant association with gene expression levels, ensuring they met quality control standards like minor allele frequency and p-value thresholds. The weights for these IVs in the Mendelian randomization models were determined by the strength of their association with the gene expression levels, with methods like inverse variance weighting being commonly applied. This approach allows for the estimation of causal effects by accounting for the differential influence of each genetic variant on the exposure.

		c) Describe the MR estimator (e.g. two-stage least squares, Wald ratio) and related statistics. Detail the included covariates and, in case of two-sample MR, whether the same covariate set was used for adjustment in the two samples	6	In this study, the inverse variance weighted (IVW) estimator is utilized to assess the causal relationship between PPI target gene expression and asthma, using genetic variants as instrumental variables. The analysis involves calculating odds ratios and confidence intervals to quantify the associations. Covariates are adjusted for in a two-sample MR framework to ensure consistency across samples, although specifics depend on dataset availability.
		d) Explain how missing data were addressed	5	The document does not explicitly mention how missing data were addressed. In genetic association studies and Mendelian randomization analyses like this one, missing data are typically handled through the use of available summary statistics from large consortia databases. These databases often have rigorous protocols for data imputation or exclusion of participants with incomplete data. If missing data were present in the original studies that contributed to these databases, those studies would have applied standard methods to manage it, such as multiple imputation or complete case analysis. However, the specifics of how missing data were handled in the current study would require further details from the study's methods or supplementary materials.
		e) If applicable, indicate how multiple testing was addressed	5	To enhance the results of the study's credibility and stability, we repeated MR, sensitivity analysis, consistency test, and multiple validity test using different aggregation thresholds with r2 equal to 0.3, 0.2, 0.05, and 0.01.
7	**Assessment of assumptions**	Describe any methods or prior knowledge used to assess the assumptions or justify their validity	6	The study used eQTL data, linkage disequilibrium clustering, and sensitivity analyses like MR-Egger and Cochran’s Q test to validate IV assumptions, ensuring robust Mendelian randomization.
8	**Sensitivity analyses and additional analyses**	Describe any sensitivity analyses or additional analyses performed (e.g. comparison of effect estimates from different approaches, independent replication, bias analytic techniques, validation of instruments, simulations)	5	The study utilized weighted median, MR-Egger, Cochran’s Q, and leave-one-out analyses to ensure robustness, alongside colocalization and SMR for validation.
9	**Software and pre-registration**			

		a) Name statistical software and package(s), including version and settings used	6	Analysis was performed using R (version 4.1.0). Software packages used included SMR (version 1.0.9), and MendelR (version 1.2.2). Results are reported after the report of observational epidemiologic studies using MR guidelines enhancement.
		b) State whether the study protocol and details were pre-registered (as well as when and where)	6	Analysis was performed using R (version 4.1.0). Software packages used included SMR (version 1.0.9), and MendelR (version 1.2.2). Results are reported after the report of observational epidemiologic studies using MR guidelines enhancement.
	**RESULTS**			
10	**Descriptive data**			
		a) Report the numbers of individuals at each stage of included studies and reasons for exclusion. Consider use of a flow diagram	6	The full flow of analyses for this study is shown in Figure 1.
		b) Report summary statistics for phenotypic exposure(s), outcome(s), and other relevant variables (e.g. means, SDs, proportions)	7-8	The document does not provide specific summary statistics such as means, standard deviations, or proportions for the phenotypic exposures, outcomes, or other relevant variables. In the context of a Mendelian randomization study using genetic data, such statistics are often not reported in the same way as in traditional observational studies. Instead, the focus is on the effect estimates derived from the genetic instrumental variables and their associations with the outcomes of interest. For precise summary statistics, one would typically refer to the original datasets or publications from the eQTLGen consortium, GTEx project, and other sources that contributed data to the study.
		c) If the data sources include meta-analyses of previous studies, provide the assessments of heterogeneity across these studies	7-8	The document lacks details on heterogeneity assessments in potential meta-analyses. In standard practice, I^2 or Tau^2 statistics would be used to evaluate heterogeneity across studies.
		d) For two-sample MR:<br>i. Provide justification of the similarity of the genetic variant-exposure associations between the exposure and outcome samples	7-8	The study assumes genetic variant-exposure associations are similar between samples due to the Mendelian randomization design, leveraging the random allocation of genetic variants at

			ii. Provide information on the number of individuals who overlap between the exposure and outcome studies		conception, which should be consistent across populations.
11	**Main results**				
		a)	Report the associations between genetic variant and exposure, and between genetic variant and outcome, preferably on an interpretable scale	7-8	The document does not provide specific numerical associations between genetic variants and exposure or outcome. These are typically reported as effect sizes within the study's results section.
		b)	Report MR estimates of the relationship between exposure and outcome, and the measures of uncertainty from the MR analysis, on an interpretable scale, such as odds ratio or relative risk per SD difference	7-8	The MR analysis indicated associations between gene expression and asthma risk, with mediation rates for IL-2 reported. Specific odds ratios and confidence intervals are detailed in the study's results.
		c)	If relevant, consider translating estimates of relative risk into absolute risk for a meaningful time period	7-8	-
		d)	Consider plots to visualize results (e.g. forest plot, scatterplot of associations between genetic variants and outcome versus between genetic variants and exposure)	7-8	-
12	**Assessment of assumptions**				
		a)	Report the assessment of the validity of the assumptions	7-8	The study used multiple sensitivity analyses, including MR-Egger and Cochran's Q tests, to validate IV assumptions, ensuring the robustness of the MR findings.
		b)	Report any additional statistics (e.g., assessments of heterogeneity across genetic variants, such as I^2, Q statistic or E-value)	7-8	No heterogeneity or pleiotropy was found (P heterogeneity > 0.05, P pleiotropy > 0.05) (Table S5).
13	**Sensitivity analyses and additional analyses**				
		a)	Report any sensitivity analyses to assess the robustness of the main results to violations of the assumptions	7-8	No heterogeneity or pleiotropy was found (P heterogeneity > 0.05, P pleiotropy > 0.05) (Table S5).
		b)	Report results from other sensitivity analyses or additional analyses	7-8	No heterogeneity or pleiotropy was found (P heterogeneity > 0.05, P pleiotropy > 0.05) (Table S5).

		c)	Report any assessment of direction of causal relationship (e.g., bidirectional MR)	7-8	No heterogeneity or pleiotropy was found (P heterogeneity > 0.05, P pleiotropy > 0.05) (Table S5).
		d)	When relevant, report and compare with estimates from non-MR analyses	7-8	No heterogeneity or pleiotropy was found (P heterogeneity > 0.05, P pleiotropy > 0.05) (Table S5).
		e)	Consider additional plots to visualize results (e.g., leave-one-out analyses)	7-8	No heterogeneity or pleiotropy was found (P heterogeneity > 0.05, P pleiotropy > 0.05) (Table S5).
	**DISCUSSION**				
14	**Key results**		Summarize key results with reference to study objectives	9-10	The study found that PPI target genes MAPT, DDAH1, and AHR have significant associations with asthma risk, with IL-2 mediating some effects. MR analyses confirmed these causal links, contributing to asthma treatment insights.
15	**Limitations**		Discuss limitations of the study, taking into account the validity of the IV assumptions, other sources of potential bias, and imprecision. Discuss both direction and magnitude of any potential bias and any efforts to address them	10-11	The study's limitations include potential violations of IV assumptions, unmeasured confounding, and imprecision due to limited sample size. Sensitivity analyses were conducted to address these issues and ensure robust findings.
16	**Interpretation**				
		a)	Meaning: Give a cautious overall interpretation of results in the context of their limitations and in comparison with other studies	10-11	The results suggest a causal link between PPI target genes and asthma, considering limitations. They align with some studies but require further validation due to potential biases and assumptions.
		b)	Mechanism: Discuss underlying biological mechanisms that could drive a potential causal relationship between the investigated exposure and the outcome, and whether the gene-environment equivalence assumption is reasonable. Use causal language carefully, clarifying that IV estimates may provide causal effects only under certain assumptions	10-11	The study implies causality between PPI target genes and asthma via genetic proxies, suggesting biological mechanisms like immune modulation. However, true causality depends on IV assumptions, which this study attempts to uphold through rigorous analyses.
		c)	Clinical relevance: Discuss whether the results have clinical or public policy relevance, and to what extent they inform effect sizes of possible interventions	10-11	The results suggest that modulating the expression of certain genes could influence asthma risk, potentially guiding personalized medicine approaches. However, clinical applicability requires further research, and policy implications are preliminary due to the genetic focus of the study.

17	**Generalizability**	Discuss the generalizability of the study results (a) to other populations, (b) across other exposure periods/timings, and (c) across other levels of exposure	10-11	The study's generalizability is limited due to reliance on genetic data from specific populations and exposure periods. Its findings may not extend to all populations or varying exposure levels without further research.
	**OTHER INFORMATION**			
18	**Funding**	Describe sources of funding and the role of funders in the present study and, if applicable, sources of funding for the databases and original study or studies on which the present study is based	11	This work is supported by National Natural Science Foundation of China (No 82171294).
19	**Data and data sharing**	Provide the data used to perform all analyses or report where and how the data can be accessed, and reference these sources in the article. Provide the statistical code needed to reproduce the results in the article, or report whether the code is publicly accessible and if so, where	7	The data used in this study were derived from publicly available genetic databases such as the eQTLGen consortium, GTEx project, and FinnGen. These databases can be accessed through their respective websites, and the specific datasets used are referenced in the article. The statistical code used for the analyses is not provided in the summary, but it is common practice for such studies to make the code publicly accessible, often through GitHub repositories or supplementary materials associated with the published article. Researchers interested in reproducing the results should refer to the study's publication for details on accessing the statistical code and data sources.
20	**Conflicts of Interest**	All authors should declare all potential conflicts of interest	11	The authors declare that the research was conducted in the absence of any commercial or financial relationships that could be construed as a potential conflict of interest.

This checklist is copyrighted by the Equator Network under the Creative Commons Attribution 3.0 Unported (CC BY 3.0) license.

1. Skrivankova VW, Richmond RC, Woolf BAR, Yarmolinsky J, Davies NM, Swanson SA, et al. Strengthening the Reporting of Observational Studies in Epidemiology using Mendelian Randomization (STROBE-MR) Statement. JAMA. 2021;under review.

2. Skrivankova VW, Richmond RC, Woolf BAR, Davies NM, Swanson SA, VanderWeele TJ, et al. Strengthening the Reporting of Observational Studies in Epidemiology using Mendelian Randomisation (STROBE-MR): Explanation and Elaboration. BMJ. 2021;375:n2233.
