# Supplementary figures and images for: Exploring the Role of Inflammation and Metabolites in Bell’s Palsy and Potential Treatment Strategies
Source: Biomedicines. 2025 Apr 13;13(4):957. doi: 10.3390/biomedicines13040957 (PMC12024589; doi:10.3390/biomedicines13040957)

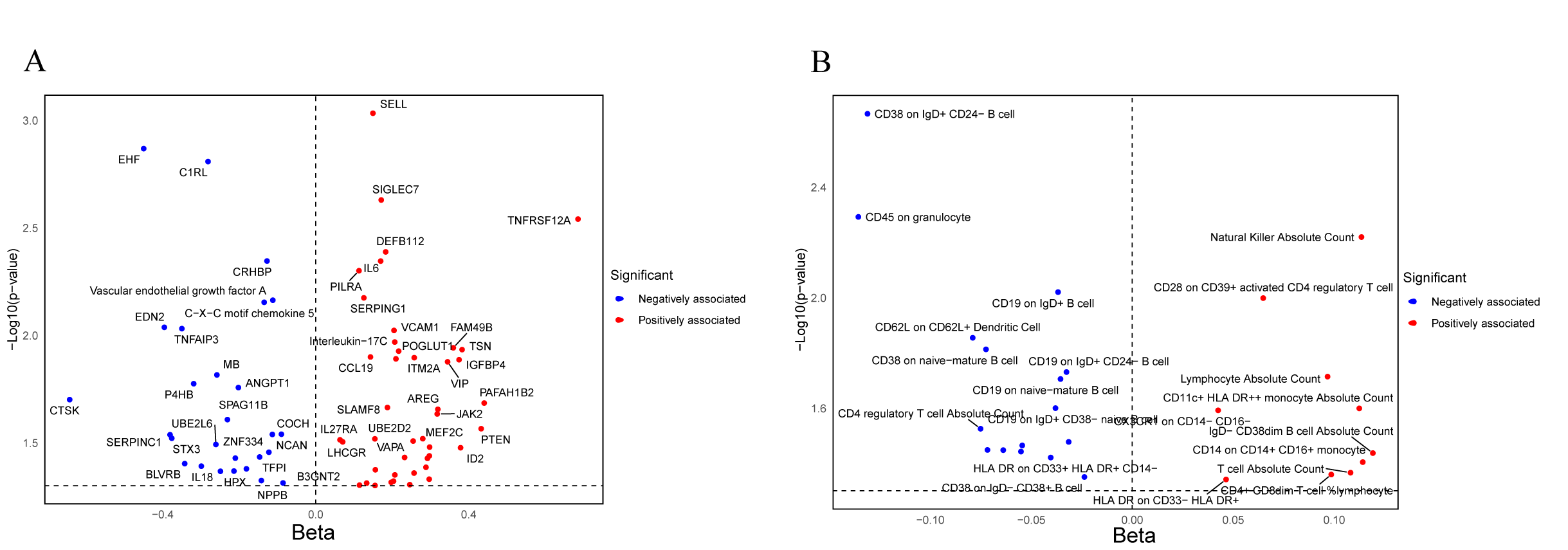

Supplement: Supplementary file 1 [file biomedicines-13-00957-s001.zip › Figure S1.tif]
